# Modeling and Simulation of Graphene-Based Transducers in NEMS Accelerometers

**DOI:** 10.3390/mi15030409

**Published:** 2024-03-18

**Authors:** Chang He, Jie Ding, Xuge Fan

**Affiliations:** 1 Advanced Research Institute of Multidisciplinary Sciences, Beijing Institute of Technology, Beijing 100081, China; 3120210697@bit.edu.cn; 2 School of Integrated Circuits and Electronics, Beijing Institute of Technology, Beijing 100081, China

**Keywords:** suspended graphene, finite element method, mechanical characteristics, NEMS devices

## Abstract

The mechanical characteristics of graphene ribbons with an attached proof mass that can be used as NEMS transducers have been minimally studied, which hinders the development of graphene-based NEMS devices. Here, we simulated the mechanical characteristics of graphene ribbons with an attached proof mass using the finite element method. We studied the impact of force, residual stress, and geometrical size on displacement, strain, resonant frequency, and fracture strength of graphene ribbons with an attached proof mass. The results show that the increase of width and thickness of graphene ribbons would result in a decrease of the displacement and strain but also an increase of resonant frequency. The increase of the length of graphene ribbons has an insignificant impact on the strain, but it could increase the displacement and decrease the resonant frequency. The increase of residual stress in the graphene ribbons decreases its strain and displacement. The estimated fracture strength of graphene shows limited dependence on its thickness, with an estimated value of around 148 GPa. These findings contribute to the understanding of the mechanical characteristics of graphene ribbons with an attached proof mass and lay the solid foundation for the design and manufacture of high-performance graphene-based NEMS devices such as accelerometers.

## 1. Introduction

Accelerometers are widely used in many areas, such as wearable motion detectors [1], the Internet of things [2], and nanoscale robotics [3]. Miniaturization is a typical trend of accelerometers in the future. However, traditional silicon-based accelerometers generally have a trade-off between sensitivity and miniaturization. Therefore, it is necessary to study the new materials for ultra-small and high-performance accelerometers.

Graphene, a two-dimensional material with a 0.335 nm monolayer thickness, was found by Konstantin Novoselov and Andre Geim in 2004 [4]. Since then, researchers have paid close attention to graphene due to its excellent mechanical and electrical properties, such as its Young’s modulus of up to 1 TPa [5,6], stretchability of up to about 20% [7], intrinsic strength of 130 GPa [5], ultra-high conductivity of 10^8^ S/m [8,9], ultra-high mobility of charge carrier (15,000 cm^2^/Vs) [10,11,12], the ability to sustain extremely high-current densities [13], atomic thickness, and the piezoresistive effect [14,15]. Therefore, graphene is a promising material for microelectromechanical systems (MEMS) and nanoelectromechanical systems (NEMS). Previous works [16,17] demonstrated that graphene can provide downscaled feasibility and higher sensitivity for NEMS sensors.

Suspended graphene with atomically thin thickness has been used as transducers to fabricate high-performance NEMS sensors for measuring pressure [18,19,20,21,22], humidity [23,24], magnetic field [25,26], gas concentration [27,28], and so on. For instance, the ultra-small and sensitive NEMS piezoresistive pressure sensor based on suspended monolayer graphene membranes was realized in 2013 [29], and the squeeze-film resonant NEMS pressure sensor based on suspended graphene was reported in 2015 [21], with a sensitivity of 9000 Hz/mbar that was 45 times higher than state-of-the art MEMS squeeze-film pressure sensors while using a 25-times-smaller membrane area. A static capacitive pressure sensor based on a suspended graphene drum was realized in 2017 [22], with the detection of capacitance change down to 50 aF and pressure differences down to 25 mbar. Suspended graphene can be used for the pirani pressure sensors, too, and the maximum relative resistance change of 2.75% between 1 and 1000 mbar and a 100-times-smaller device footprint than state-of-the-art were demonstrated in 2021 [30]. The detection of the individual physisorption of CO_2_ molecules at room temperature by using suspended double-layer graphene was reported [31]. Graphene-based high-performance hall sensors were reported, showing a linear Hall response over several hundred mT [32]. The fast and sensitive room-temperature NEMS bolometers based on focused ion-beam-structured suspended graphene were demonstrated using a resonant readout mechanism in 2019 [33].

There are few reports about suspended graphene with an attached mass [34,35,36]. The few-layer graphene cantilevers with diamond allotrope carbon masses were fabricated using focused ion-beam (FIB) deposition for studying the mechanical properties of graphene [34]. A kirigami pyramid and a variety of cantilevers based on a suspended graphene supporting 50 nm-thick gold masses were reported for actuation and the study of the mechanical properties [35]. Suspended circular graphene membranes with a mass made of either SU-8 or gold located at the center of the membrane were reported for shock detection [36]. More recently, NEMS transducers based on suspended graphene with attached proof mass for the applications of accelerometers and vibrometers have been reported [37,38,39,40]. In addition, the high-temperature accelerometer based on non-suspended graphene aerogel was reported in 2023 [41]. However, the studies of the mechanical properties and behaviors of suspended graphene with an attached proof mass in NEMS accelerometers are quite limited [36,42,43].

**Figure 1:**
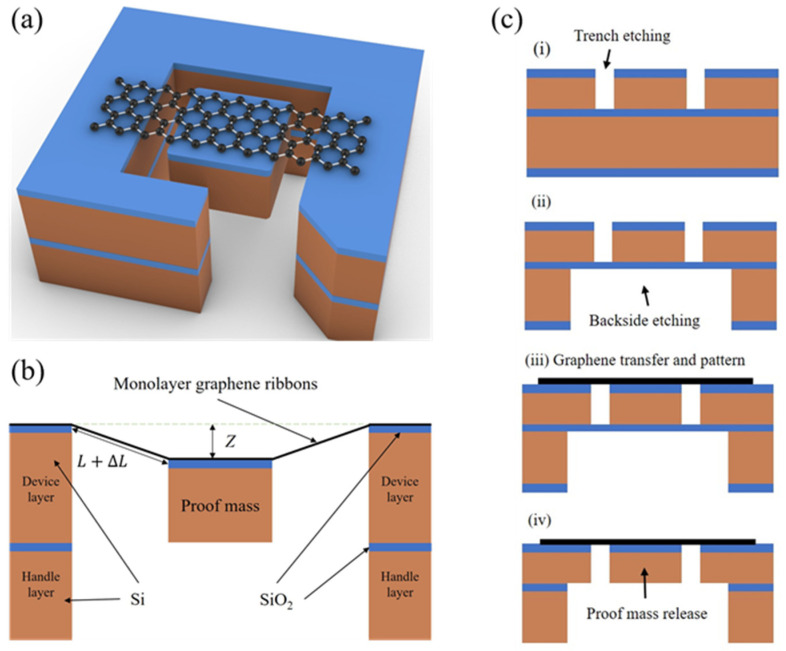
Schematic of graphene ribbons with an attached proof mass. (**a**) Three-dimensional diagram of the transducer design. (**b**) Cross-section of graphene ribbons with an attached proof mass under the applied force. (**c**) Schematic of the proposed fabrication process of graphene ribbons with an attached proof mass based on SOI (silicon on insulator): (**i**) Trench etching to define the proof mass. (**ii**) Backside etching of SOI handle layer. (**iii**) Integration of graphene with pre-fabricated SOI and patterning of the graphene. (**iv**) The release of the proof mass by etching the sacrificial layer.

In this work, we studied the structure of graphene ribbons with an attached proof mass for transducers of NEMS accelerometers provided in Figure 1a,b. By conducting a simulation using finite-element software COMSOL Multiphysics (version number: 6.0), we analyzed strain, displacement, resonant frequency, and fracture strength of such graphene transducers with different geometrical sizes, residual stresses, and forces. The proposed fabrication process of graphene ribbons with an attached proof mass was shown in Figure 1c. The results reveal mechanical characteristics of graphene ribbons with an attached proof mass and finally contribute to the understanding of NEMS accelerometers based on suspended graphene with an attached proof mass.

## 2. Results

In this paper, we built the model of graphene ribbons with an attached proof mass in COMSOL and simulated the strain, displacement, resonant frequency, and fracture strength. We have also compared the simulation results with analytical results, with the similarity of 97.5%.

The model consists of shell and solid elements, which are used for suspended graphene ribbons and the attached proof mass, respectively. The shell element, a structure mechanics module in COMSOL, is intended for mechanical characteristics of a thin-walled structure, which is the perfect representation for graphene ribbons with an attached proof mass. Additionally, graphene has a significant bending stiffness of 1.2–7.1 eV [44,45,46,47]. Therefore, we used shell instead of membrane elements in the structure mechanics module. The inevitable geometric nonlinearity of graphene ribbons should be considered as the deflection is much larger than the thickness of graphene ribbons. Thus, the stress is calculated by the Second Piola–Kirchhoff Stress Tensor, while the strain is calculated by the Green–Lagrange Strain Tensor rather than the engineering strain. The proposed transducers based on graphene ribbons with an attached proof mass could potentially be used for piezoresistive accelerometers, where the acceleration will result in the deflection of graphene ribbons and thereby change the strain and resistance of graphene ribbons. Ultra-strong van der Waals adhesion forces between SiO_2_ surface and graphene ensure that graphene ribbons can firmly attach the proof mass. The model parameters and materials are shown in Table 1. The side length of square Si/SiO_2_ proof mass is 20 μm and the height is 10 μm, with a 9 μm-thick Si layer and 1 μm-thick SiO_2_ layer.

### 2.1. Displacement

We hypothesized a rigid connection between graphene ribbons and the attached proof mass to ensure that there is no relative movement. For the much larger displacement of the proof mass attached to graphene ribbons than the thickness of the ribbon, the displacement of the attached proof mass caused by applied force can be described by [40,43]

(1) $$F=2\left[\frac{EWH^{3}}{L^{3}}\right]Z+\left[\frac{EWH}{L^{3}}\right]Z^{3}+2\left[\frac{\sigma WH}{L}\right]Z$$

where *F* is the load applied at the bottom of the proof mass, *Z* is the displacement of the attached proof mass, *E* is the Young’s modulus of the graphene, *W* is the width of the graphene ribbons, *H* is the thickness of the ribbons, *L* is the length of single side of the suspended graphene ribbons, and *σ* is the residual stress.

To verify the accuracy of our simulation, we have tested the displacement of suspended double-layer graphene ribbons using COMSOL, in which Young’s modulus was set to be 0.22 TPa, the residual stress was set to be 300 MPa, the size of proof mass of Si/SiO_2_ was set to be 40 μm × 40 μm × 16.4 μm, and the length, width, and thickness of single-side graphene ribbons were 4 μm, 6 μm, and 0.667 nm, respectively [43]. As a result, the corresponding displacement is 338 nm for the applied force of 1000 nN. Compared with the measurement result made by X. Fan in 2019 [43], in which the displacement is 374 nm under the applied force of 1000 nN and the simulation results are consistent with previous experimental results [43]. This indicates a good accuracy of our simulation.

Next, we chose defect-free graphene with the thickness of 1–10 atomic layers for the sensitive membrane of NEMS transducers. We then performed parametric simulations with a Young’s modulus of 1 TPa by applying a series of forces to study mechanical characteristics of NEMS transducers based on graphene ribbons with an attached proof mass.

**Figure 2:**
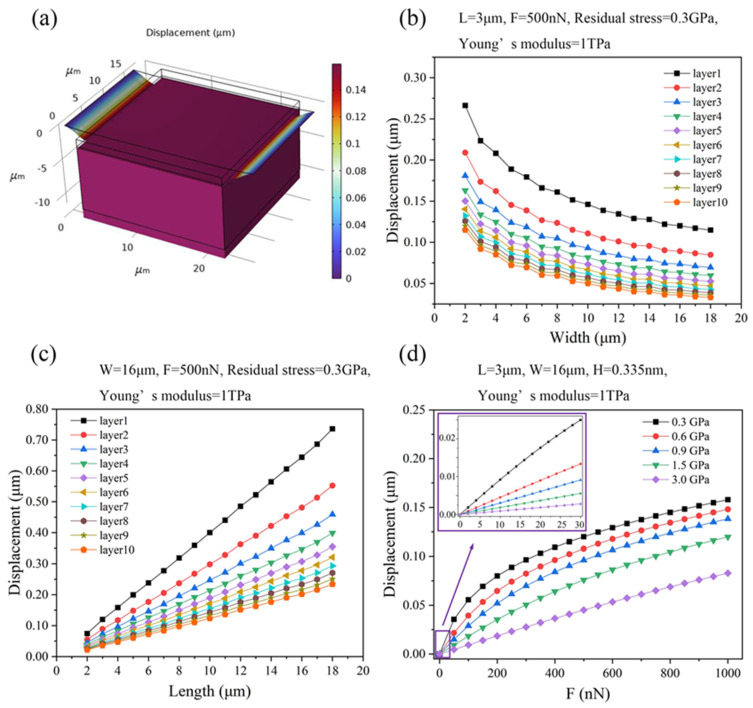
(**a**) Finite element simulation of displacement of graphene ribbons with an attached proof mass with the ribbon’s width of 16 μm and single-side ribbon’s length of 3 μm under the conditions of applied force of 1000 nN, Young’s modulus of 1 TPa, and residual stress of 0.3 GPa. (**b**) The displacement of graphene ribbons with single-side length of 3 μm and different values of thickness versus the width of graphene ribbons under the conditions of the applied force of 500 nN, Young’s modulus of 1 TPa, and residual stress of 0.3 GPa. (**c**) The displacement of graphene ribbons with the width of 3 μm and different values of thickness versus the single-side length of graphene ribbons under the conditions of the applied force of 500 nN, Young’s modulus of 1 TPa, and residual stress of 0.3 GPa. (**d**) The displacement of monolayer graphene ribbons with width of 16 μm and single-side length of 3 μm changes with the applied forces under the conditions of different values of the residual stress in the graphene ribbons.

The displacement of graphene ribbons with an attached proof mass induced by the applied force was simulated (Figure 2a). Figure 2b,c show the displacement of suspended graphene ribbons with different thickness and geometrical sizes under the conditions of the applied force of 500 nN, Young’s modulus of 1 TPa, and residual stress of 0.3 GPa. The displacement of graphene ribbons decreases by increasing the thickness and width of the graphene ribbon. In contrast, the displacement of graphene ribbons increases with the increase of the length of the graphene ribbon (Figure 2b,c). For instance, the displacement of the graphene ribbons with the width of 2 μm and single-side length of 3 μm decreases from 266 nm to 115 nm as the thickness of graphene ribbons is increased from the monolayer to 10 atomic layers (Figure 2b). The displacement of monolayer graphene ribbons with the single-side length of 3 μm decreases from 266 nm to 114 nm as the width is increased from 2 μm to 18 μm (Figure 2b). In addition, the displacement of monolayer graphene ribbons with a width of 16 μm increases from 74 nm to 736 nm as its length is increased from 2 μm to 18 μm (Figure 2c). Further, as shown in Figure 2b,c, compared with the width of graphene ribbons, the length of graphene ribbons has a prominent impact on the displacement of graphene ribbons.

Figure 2d shows how the displacement of monolayer graphene ribbons with a width of 16 μm and single-side length of 3 μm changes with applied forces under the conditions of different values of the residual stress in the graphene ribbons. The displacement of graphene ribbons decreases with the increase of the residual stress while the displacement of graphene ribbons increases with the increase of the applied force. For instance, as the residual stress is increased from 0.3 GPa to 3 GPa, the displacement of graphene ribbons decreases from 120 nm to 45 nm at the applied force of 500 nN. As the force is increased from 0 nN to 1000 nN, the displacement of graphene ribbons increases from 0 nm to 158 nm at the residual stress of 0.3 GPa.

Further, as shown in Figure 2d, as the applied force is small, the cubic term $\left[\frac{EWH}{L^{3}}\right]Z^{3}$ in Equation (1) can be negligible compared with the residual tension term $2\left[\frac{\sigma WH}{L}\right]Z$, so the relationship between displacement and force is observed to be linear (the inset of Figure 2d). As the applied force increases and is large enough, the cubic term $\left[\frac{EWH}{L^{3}}\right]Z^{3}$ in Equation (1) will be larger than the linear terms $2\left[\frac{EWH^{3}}{L^{3}}\right]Z+2\left[\frac{\sigma WH}{L}\right]Z$, resulting in a nonlinear power function relationship between displacement and applied force.

### 2.2. Strain

**Figure 3:**
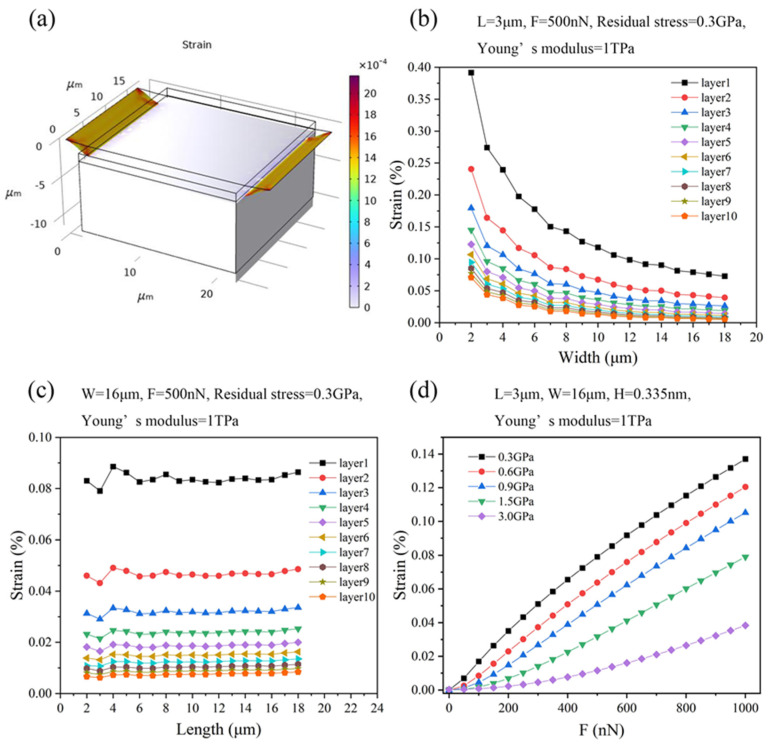
(**a**) Finite element simulation of strain of graphene ribbons with an attached proof mass with the ribbon’s width of 16 μm and single-side ribbon’s length of 3 μm under the conditions of the applied force of 1000 nN, Young’s modulus of 1 TPa, and residual stress of 0.3 GPa. (**b**) The strain of graphene ribbons with single-side length of 3 μm and different values of thickness versus the width of graphene ribbons under the conditions of the applied force of 500 nN, Young’s modulus of 1 TPa, and residual stress of 0.3 GPa. (**c**) The strain of graphene ribbons with the width of 3 μm and different thickness versus the single-side length of graphene ribbons under the conditions of the applied force of 500 nN, Young’s modulus of 1 TPa, and residual stress of 0.3 GPa. (**d**) The strain of monolayer graphene ribbons that have the width of 16 μm and single-side length of 3 μm changes with the applied forces under the conditions of different values of the residual stress in the graphene ribbons.

The force applied on the suspended graphene ribbons results in deflection and, therefore, strain, causing a change in the resistance of graphene ribbons. We simulated the strain in the graphene ribbons under the conditions of different thicknesses, lengths, widths, residual stresses of graphene ribbons, and different forces acting on the suspended graphene ribbons (Figure 3a).

Figure 3b,c shows the strain of suspended graphene ribbons with different thicknesses and geometrical sizes under the conditions of the applied force of 500 nN, Young’s modulus of 1 TPa, and residual stress of 0.3 GPa. The strain of graphene ribbons decreases with increasing graphene ribbon thickness and width (Figure 3b,c). However, the strain of graphene ribbons almost does not depend on the length of graphene ribbons (Figure 3c), which is consistent with the previous theoretical derivation [43]. For instance, the strain of graphene ribbons with the width of 2 μm and single-side length of 3 μm decreases from 0.39% to 0.07% as the thickness of graphene ribbons is increased from a monolayer to 10 atomic layers (Figure 3b). The strain of the monolayer graphene ribbons with single side length of 3 μm decreases from 3.9% to 0.073% as the width of graphene ribbons increases from 2 μm to 18 μm (Figure 3b).

Figure 3d shows how the strain of monolayer graphene ribbons with a width of 16 μm and single-side length of 3 μm changes with applied forces under the conditions of different values of the residual stress in the graphene ribbons. The strain of graphene ribbons decreases with the increase of residual stress, while it increases with the increase of the applied force. For instance, as the residual stress is increased from 0.3 GPa to 3 GPa, the strain of graphene ribbons decreases from 0.08% to 0.011% at the applied force of 500 nN. As the force is increased from 0 nN to 1000 nN, the strain of graphene ribbons increases from 0 to 0.13% at the residual stress of 0.3 GPa. Further, as shown in Figure 3d, the strain has an approximate linear relationship with the applied force, which is the benefit of piezoresistive sensors based on graphene ribbons. The strain could be described by Equation (2) [43] when the cubic term $\left[\frac{EWH}{L^{3}}\right]Z^{3}$ of Equation (1) is much larger than the linear terms $2\left[\frac{EWH^{3}}{L^{3}}\right]Z+2\left[\frac{\sigma WH}{L}\right]Z$ in Equation (1).

(2) $$\varepsilon =\frac{∆L}{L}\approx \frac{Z^{2}}{{2L}^{2}}\approx \;\frac{1}{2}{\left(\frac{F}{EWH}\right)}^{\frac{2}{3}}$$

### 2.3. Resonant Frequency

The force acting on the suspended graphene ribbons results in stress that builds up in the ribbons, hence causing a change in the resonant frequency of the spring–mass system, which is provided by Equation (3) [39] where *f* is the resonant frequency, k is the spring constant of the linear system, and m is the mass of the spring–mass system.

(3) $$f=\frac{1}{2\pi }\sqrt{\frac{k}{m}}\;=\frac{1}{2\pi }\sqrt{\frac{1}{m}(2\left[\frac{EWH^{3}}{L^{3}}\right]+3\left[\frac{EWH}{L^{3}}\right]Z^{2}+2\left[\frac{\sigma WH}{L}\right]})$$

**Figure 4:**
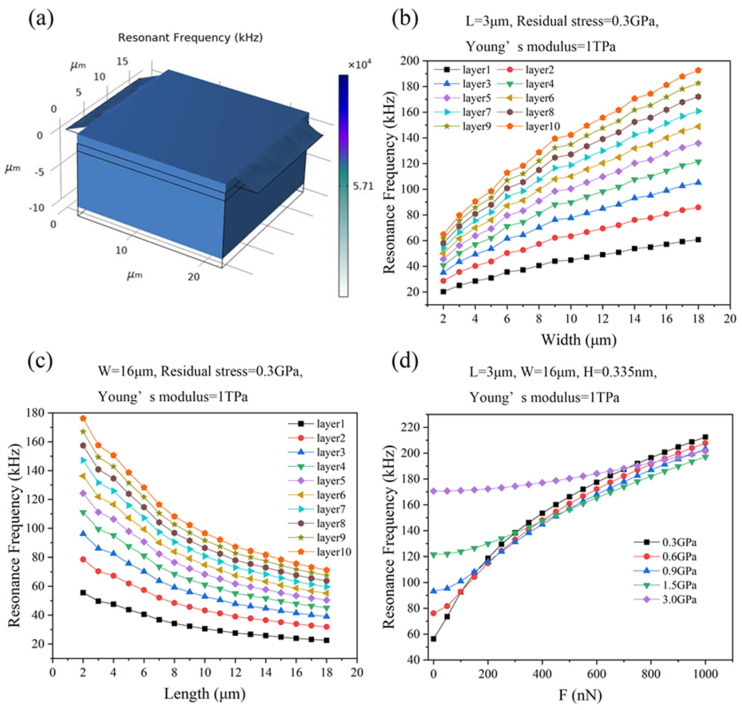
(**a**) Finite element simulation of resonant frequency of graphene ribbons with an attached proof mass with the ribbon’s width of 16 μm and single-side ribbon’s length of 3 μm under the conditions of the applied force of 1000 nN, Young’s modulus of 1 TPa, and residual stress of 0.3 GPa. (**b**) The resonant frequency of graphene ribbons with an attached proof mass with single-side ribbon length of 3 μm and different values of thickness of graphene ribbons versus the width of the graphene ribbon under the conditions of the applied force of 500 nN, Young’s modulus of 1 TPa, and residual stress of 0.3 GPa. (**c**) The resonant frequency of graphene ribbons with an attached proof mass with the ribbon’s width of 3 μm and different values of thickness of the graphene ribbons versus the single-side length of graphene ribbons under the conditions of the applied force of 500 nN, Young’s modulus of 1 TPa, and residual stress of 0.3 GPa. (**d**) The resonant frequency of monolayer graphene ribbons with ribbon’s width of 16 μm and single-side ribbon’s length of 3 μm changes with the applied forces under the conditions of different values of the residual stress in the graphene ribbons.

We simulated the resonant frequency of the graphene ribbons with an attached proof mass under the conditions of different thicknesses, single-side lengths, widths, residual stresses of graphene ribbons, and different applied forces acting on the suspended graphene ribbons (Figure 4a).

Figure 4b,c shows the resonant frequency of graphene ribbons with different thicknesses and geometrical sizes under the conditions of the applied force of 0 nN, Young’s modulus of 1 TPa, and residual stress of 0.3 GPa. The resonant frequency of graphene ribbons with an attached proof mass increases with the increase of the thickness of the graphene ribbons, as well as the width of graphene ribbons, while it decreases with the increase of the length of graphene ribbons (Figure 4b,c). For instance, the resonant frequency of graphene ribbons with an attached proof mass with the ribbon’s width of 2 μm and single-side ribbon’s length of 3 μm increases from 20.22 kHz to 64.91 kHz as the thickness of graphene ribbons increases from the monolayer to 10 atomic layers (Figure 4b). The resonant frequency of the monolayer graphene ribbons with an attached proof mass with the single-side ribbon’s length of 3 μm increases from 20.22 kHz to 60.60 kHz as the width of graphene ribbons is increased from 2 μm to 18 μm (Figure 4b). In addition, the resonant frequency of monolayer ribbons with an attached proof mass with a ribbon’s width of 16 μm decreases from 55.54 kHz to 22.53 kHz as its length is increased from 2 μm to 18 μm (Figure 4c).

Figure 4d shows how the resonant frequency of monolayer graphene ribbons with an attached proof mass with the ribbon’s width of 16 μm and the single-side ribbon’s length of 3 μm changes with the applied forces under the conditions of different values of the residual stress in the graphene ribbons. The resonant frequency increases with the applied force, ranging from tens of kHz to hundreds of kHz. This indicates that graphene ribbons with an attached proof mass can potentially be used as resonant sensors such as accelerometers and vibration sensors [39,43,48].

As the residual stress was large enough, such as 1.5 GPa or 3 GPa, the resonant frequency has relatively weak dependence on the applied force compared with small residual stresses such as 0.3 GPa and 0.6 GPa. Further, as the applied force was 0 nN and the residual stresses were 0.3 GPa, 0.6 GPa, 0.9 GPa, 1.5 GPa, and 3 GPa, the resonant frequencies of graphene ribbons with an attached proof mass were 56.30 kHz, 76.19 kHz, 93.19 kHz, 121.44 kHz, and 170.61 kHz, respectively. As the applied force was small, it is the magnitude of the residual stress that mainly determines the value of the resonant frequency. That is, the larger the residual stress in the graphene ribbons, the higher the resonant frequency of graphene ribbons with an attached proof mass. With the increase of the applied force, the impact of the applied force on the resonant frequency is more and more significant compared to the impact of residual stress on the resonant frequency. For instance, as the applied force is increased to about 400 nN, the resonant frequency of graphene ribbons with an attached proof mass that have low residual stress (less than 0.9 GPa) is larger than that of graphene ribbons with an attached proof mass that have residual stress of 1.5 GPa. Further, as the applied force is increased to about 800 nN, the resonant frequency of graphene ribbons with an attached proof mass that have low residual stress (less than 0.9 GPa) is larger than that of graphene ribbons with an attached proof mass that have a residual stress of 3 GPa.

### 2.4. Fracture Strength

**Figure 5:**
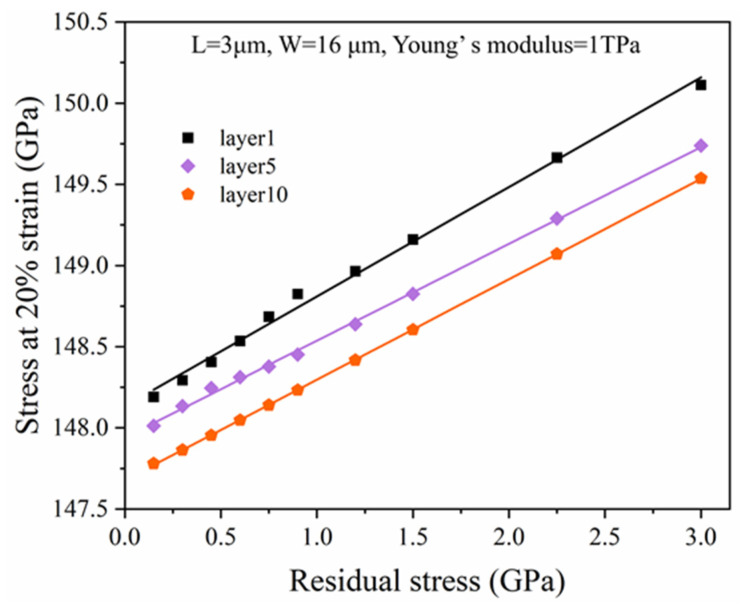
Stress at 20% strain of one, five, and 10 atomic layer-thick graphene ribbons with an attached proof mass with residual stresses of graphene ribbons ranging from 0.15 GPa to 3 GPa and ribbon’s width of 16 μm and single-side ribbon’s length of 3 μm.

The fracture strength of the graphene is generally defined by the maximum loading force divided by its cross-section before the graphene ribbon rupture; it is not affected by the geometrical size of graphene ribbons according to its definition. We hypothesized that the maximum strain of graphene is 20% [49]. Therefore, we estimated the fracture strength of graphene by simulating the stress at a 20% strain of one, five, and 10 atomic layer-graphene ribbons with an attached proof mass, assuming residual stresses of graphene ribbons ranging from 0.15 GPa to 3 GPa, a ribbon’s width of 16 μm, and a single-side ribbon’s length of 3 μm. As shown in Figure 5, the stress at 20% strain of graphene ribbons with an attached proof mass increases with the increase of residual stress. While the stress at 20% strain of graphene ribbons with an attached proof mass is almost unaffected by the increased thickness of graphene ribbons. According to the fitting curve, as the thickness of graphene ribbons were one, five, and 10 atomic layers, the stress at 20% strain without residual stress is around 148.13 GPa, 147.94 GPa, and 147.68 GPa, respectively. This indicates that the fracture strength of graphene almost does not depend on the thickness of the graphene ribbons, with a slight decrease with the increase of the thickness. Compared with the fracture strength of 130 GPa [5], 110–140 GPa [49], and 175 GPa [50], our simulation result is close to those based on existing experiments and calculations.

## 3. Conclusions

The purpose of this work was to comprehensively study the impact of force, residual stress, and geometrical size of graphene ribbons on the mechanical characteristics of transducers based on graphene ribbons with an attached proof mass. We performed COMSOL FEA simulation to achieve and analyze the displacement, strain, resonant frequency, and fracture strength of graphene ribbons with an attached proof mass. We found the increase of the width or thickness of graphene ribbons would result in the decrease of both the displacement and strain of graphene ribbons. But the increase of the width or thickness of graphene ribbons would result in the increase of resonant frequency. The increase of length of graphene ribbons has an insignificant impact on the strain of graphene ribbons but could increase the displacement of graphene ribbons and decrease the resonant frequency. Further, we also found that the increase of residual stress in the graphene ribbons could decrease the strain and displacement of the graphene ribbons. The fracture strength of graphene almost does not depend on the thickness of the graphene ribbons, with an estimated value of around 148 GPa.

Compared with other simulation work about graphene-based accelerometers such as the design of the accelerometer based on graphene nanoribbon resonators without proof mass [51] and the design of the accelerometer based on graphene resonators with the Au proof mass (the size: 30 nm × 30 nm × 30 nm) [52], the proof mass that is suspended by the graphene ribbons in this work is orders of magnitude larger. Such larger proof mass would potentially have higher sensitivity as it is designed and used in the transducers based on suspended graphene with an attached proof mass for the application of accelerometers. Compared with other types of accelerometers such as silicon-based accelerometers, graphene-based accelerometers could potentially reduce the size of the sensors and, at the same time, substantially increase the sensitivity.

These findings would contribute to the understanding of the mechanical characteristics of transducers based on graphene ribbons with an attached proof mass and would lay the solid foundation for the design and fabrication of high-performance graphene NEMS sensors, such as accelerometers, vibration sensors, and gyroscopes.

## Figures and Tables

**Table 1:** The parameters of graphene and SiO_2_/Si proof mass.

Materials	Young’s Modulus (GPa)	Poisson’s Ratio	Density (kg/m^3^)
Suspended graphene	1000	0.16	2250
SiO_2_ layer	71	0.17	2200
Si layer	170	0.28	2330

## Data Availability

Data are contained within the article.
